# Risk Factors and Outcomes for Invasive Infection Among Patients Colonized with *Candidozyma auris*: A Case–Control Study

**DOI:** 10.3390/antibiotics14121206

**Published:** 2025-12-01

**Authors:** Shuroug A. Alowais, Haytham A. Wali, Khalid bin Saleh, Rema Aldugiem, Yara Alsaeed, Meshari Almutairi, Thamer A. Almangour, Yazed S. Alsowaida, Mohammad Bosaeed

**Affiliations:** 1 College of Pharmacy, King Saud bin Abdulaziz University for Health Sciences, Riyadh 14611, Saudi Arabiaaldugiem.ra@gmail.com (R.A.); yara.ahmedals@gmail.com (Y.A.); mesharibandar@gmail.com (M.A.); 2 King Abdullah International Medical Research Center, Riyadh 11481, Saudi Arabia; bosaeedmo@kaimrc.edu.sa; 3 King Abdulaziz Medical City, National Guard Health Affairs, Riyadh 11426, Saudi Arabia; 4 Department of Pharmacy Practice, College of Clinical Pharmacy, King Faisal University, Al-Ahsa 31982, Saudi Arabia; hwali@kfu.edu.sa; 5 Department of Clinical Pharmacy, College of Pharmacy, King Saud University, Riyadh 11451, Saudi Arabia; talmangour@ksu.edu.sa; 6 Department of Clinical Pharmacy, College of Pharmacy, University of Ha’il, Ha’il 55473, Saudi Arabia; ysowaida@gmail.com; 7 College of Medicine, King Saud bin Abdulaziz University for Health Sciences, Riyadh 14611, Saudi Arabia

**Keywords:** *Candidozyma auris*, colonization, invasive infection, fungal infection, risk factors

## Abstract

Background: *Candidozyma auris* has emerged globally as a multidrug-resistant pathogen with high rates of colonization and potential for invasive infection. Understanding the progression from colonization to infection and associated outcomes is limited. This study aimed to describe the characteristics and outcomes of *C. auris*-colonized patients and identify risk factors for progression to infection. Methods: We conducted a single-center retrospective case–control study of hospitalized patients with documented *C. auris* colonization at a tertiary care center (2018–2024). Cases were patients who progressed to a fungal infection (infection group), while controls remained colonized (colonized group). Demographics, comorbidities, device use, prior antimicrobial exposure, microbiologic characteristics, and outcomes were compared. Results: Among 334 *C. auris*-colonized patients, 44 (13.2%) progressed to infection. The median age was 66.4 years. Those in the infection group had higher Charlson Comorbidity Index (5.9 ± 3.1 vs. 4.79 ± 2.7, *p* = 0.0112) and were more likely to have central line (90.9% vs. 71.7%, *p* = 0.0067), mechanical ventilation (77.3% vs. 60.34%, *p* = 0.0306), parenteral nutrition (6.82% vs. 1.72%, *p* = 0.0395), and abdominal surgery (13.64% vs. 4.83%, *p* = 0.0217). In multivariate analysis, abdominal surgery was the only independent predictor of infection (OR 4.08; 95% CI 1.1–15.1; *p* = 0.03). In-hospital mortality was significantly higher in the infection group (65.9% vs. 33.1%, *p* < 0.0001). Conclusions: Approximately one in eight colonized patients developed *C. auris* infection. Recent abdominal surgery independently predicted progression, while infection was associated with higher mortality and prolonged hospitalization, underscoring the need for targeted prevention in high-risk patients.

## 1. Introduction

*Candidozyma auris* is an emerging multidrug-resistant (MDR) fungal pathogen first identified in 2009 from the external ear canal of a patient in Japan [1]. Since then, it has been reported worldwide as a cause of invasive infections, particularly bloodstream infections, and has posed significant challenges to healthcare systems due to its resistance to multiple antifungal agents and its ability to cause outbreaks [2]. The World Health Organization has classified *C. auris* as a critical priority pathogen, underscoring its global health threat [3]. Per the CDC, since *C. auris* was first reported in 2016, the reported *C. auris* clinical cases have continued to increase [4], and the UK Health Security Agency’s most updated report from August 2024 states that *C. auris* has been identified across six continents, in more than 60 countries worldwide [5]. Outbreaks have been reported across healthcare facilities, particularly in intensive care units (ICUs), where *C. auris* can persist on surfaces and equipment, facilitating the transmission of nosocomial infections [6].

In Saudi Arabia, the first confirmed cases were reported in 2017–2018 in Riyadh and Dammam, with isolates belonging to the South Asian clade [7]. Subsequent reports from Riyadh and Makkah have described additional cases, often in critically ill patients with multiple comorbidities and prolonged hospital and ICU stays [8,9]. Globally, *C. auris* is divided into several geographically and genetically distinct clades, most commonly recognized as clade I (South Asian), clade II (East Asian), clade III (African), clade IV (South American), and a fifth (clade V) initially identified in Iran [10,11]. In Saudi Arabia, recent genomic data show that isolates are mostly in clade I (South Asian) [6]

Management of *C. auris* is complicated by resistance to all three major antifungal drug classes in some isolates, the emergence of pan-resistant strains, and frequent misidentification, as many conventional biochemical and phenotypic identification systems (e.g., API 20C, Vitek 2, Phoenix, MicroScan) often misidentify *C. auris* as related species such as *C. haemulonii*, *C. duobushaemulonii*, or *C. lusitaniae*. These challenges stem from the limited ability of routine laboratory platforms to differentiate closely related Candida species. Accurate identification requires matrix-assisted laser desorption ionization–time of flight (MALDI-TOF) with updated databases or molecular methods such as PCR or sequencing [12,13]. Effective control requires accurate laboratory identification, antifungal susceptibility testing, strict infection prevention measures, and ongoing surveillance.

Colonization is defined as the presence of *C. auris* on body sites such as the skin, nares, axilla, or groin without clinical illness [14]. Colonized individuals can remain reservoirs for extended periods, often several months, thereby contributing to the persistence and transmission of the infection [15,16]. In contrast, invasive infection occurs when *C. auris* is introduced into sterile sites such as the bloodstream or cerebrospinal fluid, leading to severe illness with high morbidity and mortality [2,17]. Approximately 5–10% of colonized patients develop invasive infection, with risk exceeding 25% among critically ill patients within 60 days of detection [18,19,20].

Although risk factors for invasive Candida infections in general, such as invasive medical devices, broad-spectrum antimicrobial exposure, immunosuppression, prolonged ICU stay, and recent surgery, are well described [19,20,21], few studies have specifically examined predictors of progression from *C. auris* colonization to infection. Existing evidence, mostly from outside the Middle East, suggests potential associations with total parenteral nutrition, colonization at multiple sites, prior antifungal therapy, advanced kidney disease, higher APACHE II scores, and sepsis [22,23,24,25,26]. However, data from Saudi Arabia remains limited, and regional differences in patient characteristics, antimicrobial use, and infection control practices may influence risk.

To address this gap, the present study aims to describe the clinical characteristics and outcomes of hospitalized patients colonized with *C. auris* at a tertiary care center in Saudi Arabia, compare those who progressed to invasive infection with those who did not, and identify risk factors for progression.

## 2. Results

### 2.1. Study Population and Baseline Characteristics

**Figure 1:**
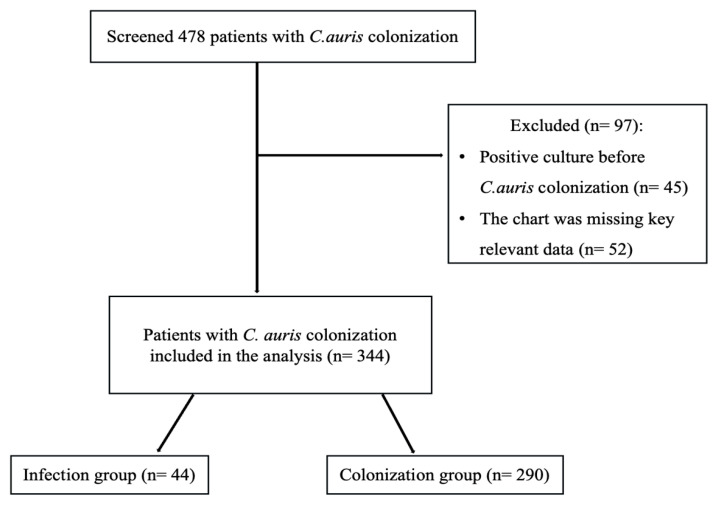
Flow diagram for the inclusion and exclusion of the study cohort.

A total of 478 patients with documented positive *C. auris* colonization were screened. Of these, 162 patients were excluded because of either a positive *C. auris* culture prior to colonization or missing key relevant data (Figure 1). The final cohort comprised 334 patients, of whom 44 (13.2%; 95% confidence interval [CI], 0.0994 to 0.1725) progressed to invasive infection (infection group), and 290 (86.8%) remained colonized without infection (colonization group).

The mean age was similar between the colonization and infection groups (66.13 ± 16.96 vs. 68.20 ± 15.61 years, *p* = 0.4467). The proportion of men was also comparable between the two groups (57.2% vs. 54.5%, *p* = 0.7365). Laboratory inflammatory markers at the time of colonization, including white blood cell (WBC) count, C-reactive protein (CRP) levels, and procalcitonin levels, did not differ significantly between the colonization and progressed infection groups.

The Charlson Comorbidity Index was significantly higher in the infection group than in the colonization group (5.9 ± 3.1 vs. 4.79 ± 2.7, *p* = 0.0112). Diabetes mellitus and moderate to severe chronic kidney disease (CKD) were more common in the infection group compared to the colonization group, though the difference was not statistically significant.

Multi-site colonization was identified in nearly half of the patients in both groups, with no significant difference. There were no statistically significant differences in the colonization source between the two groups. Central venous catheter use was significantly more frequent in the infection group (90.9%) than in the colonization group (71.7%; *p* = 0.0067). Similarly, mechanical ventilation was significantly more common among the infection group (77.3% vs. 60.34%, *p* = 0.0306). Use of total parenteral nutrition (TPN) within 30 days of colonization was more frequent in the infection group (6.82% vs. 1.72%, *p* = 0.0395). Similarly, abdominal surgery within 30 days was more common in the infection group (13.64% vs. 4.83%, *p* = 0.0217); both variables are statistically significant. The complete baseline characteristics for the study cohort are presented in Table 1.

### 2.2. Microbiologic and Temporal Characteristics of C. auris Infection

Among the 44 patients in the infection group, the median time from the initial colonization to the first positive *C. auris* culture was 21 days [IQR: 12–35]. The most common source of *C. auris* isolation was urine, identified in 25 patients (56.82%), followed by bloodstream infections in 19 patients (43.18%). Antifungal susceptibility testing was performed in 20 isolates (45.45%), revealing echinocandin resistance in only a minority of cases. Specifically, resistance to anidulafungin was detected in 1 patient (5%) and to caspofungin in 3 patients (15%) (Table 2).

### 2.3. Treatment and Outcomes Post C. auris Colonization

In-hospital mortality was significantly higher in the infection group than in the colonization group (65.9% [n = 29] vs. 33.1% [n = 96]), with an odds ratio (OR) of 3.91 (95% confidence interval [CI]: 2.0–7.6; *p* = 0.0001). In contrast, 30-day mortality rates were comparable between the two groups: 25% (n = 11) in the infection group and 23.79% (n = 69) in the colonization group (OR: 1.07, 95% CI: 0.51–2.22, *p* = 0.8613).

The median time to death was significantly longer in the infection group (43 days [IQR: 25–88]) than in the colonization group (15 days [IQR: 5–40]; *p* = 0.000233). Patients in the infection group also had a significantly more extended median ICU stay (30 days [IQR: 10–66] vs. 17 days [IQR: 7–36]; *p* = 0.03196) and more extended hospital stay overall (73 days [IQR: 43–147] vs. 40 days [IQR: 20–83]; *p* = 0.000446) (Table 3).

### 2.4. Risk Factors for Progression from Colonization to Infection

Progression from *C. auris* colonization to *C. auris* infection was significantly associated with a history of abdominal surgery within the preceding 30 days (OR 4.08; 95% CI: 1.10–15.12; *p* = 0.03). In-hospital mortality was also significantly higher among patients who developed infection (OR 7.80; 95% CI: 3.28–18.50; *p* < 0.001). Other factors, including the presence of a central venous catheter, mechanical ventilation, and receipt of TPN within 30 days, were not significantly associated with progression. A summary of the logistic regression analysis of risk factors is presented in Table 4.

## 3. Discussion

In this retrospective case–control study, we evaluated the characteristics, risk factors, microbiologic data, and outcomes associated with *C. auris* colonization and progression to infection. We found that approximately 13% of patients colonized with *C. auris* developed invasive *C. auris* infection. Patients in the infection group had higher Charlson Comorbidity Index scores. They were more likely to have a central venous catheter, receive mechanical ventilation or TPN, or undergo abdominal surgery within 30 days of colonization. Compared with the colonization group, those in the infection group experienced higher in-hospital mortality as well as longer hospital and ICU stays. These findings provide novel insight into the epidemiology and clinical burden of *C. auris* colonization and infection in a Middle Eastern healthcare setting over 6.5 years.

In our cohort, the overall rate of *C. auris* infection was 13.2%. Among these cases, less than half were bloodstream infections, representing 5.7% of the total cohort, while the remaining 7.5% were classified as UTI cases. Reported progression rate from the date of initial *C. auris* colonization to invasive infection varies widely in the literature, ranging from approximately 4% to 57%. For instance, a New York cohort study found that 4% of 187 patients colonized developed bloodstream infection, with a median time of 86 days from colonization to candidemia, which is quite comparable to the interval in our study [19]. In contrast, national data from Saudi Ministry of Health hospitals reported a substantially higher progression rate of 56.9% among 511 colonized patients, although the time from colonization to infection was not specified [29]. Moreover, a single-center retrospective study of critically ill ICU patients noted that 17% of 157 colonized individuals progressed to candidemia, with a cumulative risk exceeding 25% within 60 days [20], which is higher than what is reported in our study. More recently, Park et al. reported that among 97 colonized or infected patients, 31 (32%) had invasive infection, and bloodstream infection accounted for 29% of these cases (9/31), corresponding to 9.3% of the total cohort [26]. Overall, our findings fall within the established range reported in the literature, emphasizing the considerable risk of invasive infections following documented colonization.

A key finding in our study was that abdominal surgery within 30 days of colonization was a significant independent risk factor associated with progression to invasive *C. auris* infection in the logistic regression model. This association may reflect disruption of mucosal barriers, exposure of intra-abdominal sites, and increased need for invasive devices and broad-spectrum antibiotics postoperatively, all of which can facilitate fungal invasion. Similar findings have been reported in other Candida species infections, where gastrointestinal or surgical procedures have been identified as significant risk factors for candidemia [23,24,25]. To our knowledge, this is the first large cohort study from Saudi Arabia to demonstrate abdominal surgery as an independent predictor of progression from colonization to infection, highlighting a practical target for early monitoring and intervention in postoperative patients.

Notably, WBC count, CRP, and procalcitonin did not differ significantly between patients who progressed to infection and those who remained colonized. This finding is explained by the fact that inflammatory markers in our study were collected at the time of colonization detection for all patients, including those who later developed invasive infection. Because these patients were not yet infected when these values were obtained, elevations in inflammatory markers would not be expected. Therefore, these results reflect baseline clinical status rather than inflammatory response to invasive *C. auris* infection.

An essential factor influencing progression to invasive infection is the high fungal burden associated with *C. auris*. Clinical studies have shown that higher colonization densities are associated with an increased risk of invasive infection [30,31]. Furthermore, *C. auris* forms dense biofilms and strongly adheres to both skin and abiotic surfaces, enabling long-term persistence despite standard infection control practices [30,31]. These biofilms serve as a reservoir for bloodstream invasion, allowing the organism to survive in deeper skin layers and medical devices, and they also enhance antifungal resistance [30]. Moreover, *C. auris* possesses several immune-evasion mechanisms, including reduced recognition by innate immune receptors, resistance to neutrophil and macrophage killing, and tolerance to oxidative and nitrosative stress [30]. The literature also suggests that *C. auris* employs immune-evasion mechanisms mediated by cell-wall remodeling, particularly mannan-mediated masking of β-glucan. The pathogen has a dense outer mannan layer in its cell wall, which conceals the inner β-glucan structures from host pattern recognition receptors (PRRs). This allows *C. auris* to evade key innate immune receptors, including Dectin-1 on phagocytes, leading to neutrophils with minimal phagocytic activity [31]. Another notable immune-evasion mechanism of *C. auris* is its ability to modulate the IL-1R pathway. This modulation represents a complex strategy in which *C. auris* manipulates host cytokine regulation by inducing the IL-1 receptor antagonist (IL-1Ra), thereby suppressing IL-1 receptor signaling and impairing key pro-inflammatory functions [32]. Overall, these characteristics increase the likelihood of progression from colonization to invasive infection.

The majority of patients in both the infection and colonization groups were elderly. Patients in the infection group experienced significantly worse clinical outcomes, including higher in-hospital mortality and longer ICU and hospital stays. Notably, the Charlson Comorbidity Index was significantly higher, indicating a greater burden of chronic illness as a potential driver of infection risk. Similarly, a recent study conducted in Saudi Arabia reported that age and comorbidities are strong predictors of *C. auris* infection [33], consistent with our findings and underscoring the role of host-related factors in disease progression.

The presence of invasive devices, particularly central venous catheters and mechanical ventilation, was significantly higher in the infection group, particularly in ICU settings. Our results are consistent with a study reporting that all patients in the first Venezuelan outbreak of *C. auris* candidemia had central venous catheters before the infection [34]. Our findings are further supported by a recent study in Saudi Arabia, which found that the presence of indwelling medical devices, such as catheters or central lines, at admission was significantly associated with an increased risk of *C. auris* colonization or infection [33]. In contrast, another study described the frequent use of urinary catheters, tracheostomy tubes, and mechanical ventilation among affected patients, with no independent association found between these interventions and infection [35]. Consistent with these findings, our logistic regression analysis showed that central venous catheterization and mechanical ventilation were not independently associated with progression from colonization to infection, suggesting that these factors may reflect overall illness severity rather than direct causal contributors.

Despite expanding research on *C. auris* infections, data concerning the risk factors contributing to the progression from colonization to invasive infection are insufficient. Prior studies have proposed multiple risk factors, including prolonged antibiotic or antifungal exposure, critical illness, TPN, and medical comorbidities [22,23,24,25,26]. The use of broad-spectrum antibiotics was common in our colonized cohort; the pattern of antibiotic use may contribute to microbiome disruption and fungal overgrowth. Our results are consistent with the current literature. A cross-sectional study was conducted to investigate the significant risk factors associated with the development of *C. auris* infection. Notably, they found that prior use of broad-spectrum antibiotics was identified in 22% of patients infected or colonized with *C. auris* [33]. Another study showed that all patients with *C. auris* candidemia had a history of prior antibiotic exposure [34]. In contrast, a different study identified prior antifungal use as a primary predictor of *C. auris* infection [35]. These findings emphasize that previous exposure to antibiotics and antifungals is a significant risk factor that facilitates the emergence of fungi.

In-hospital mortality was significantly higher in the infection group compared to the colonization group, indicating a strong association between infection and increased risk of death during hospitalization. In our logistic regression model, infection was independently associated with a markedly increased odds of in-hospital mortality, underscoring the substantial clinical burden of invasive *C. auris* infection. These findings align with previous studies reporting in-hospital mortality rates ranging from 30 to 80% in patients with invasive *C. auris* infections [2,36,37,38,39]. For instance, Chowdhary et al. found that critically ill patients with COVID-19 and positive Candida blood cultures had a high case-fatality rate of approximately 60%. Similarly, our cohort demonstrated an in-hospital mortality rate of around 66% following a positive *C. auris* infection, highlighting the high mortality burden of this pathogen across different cohorts [36].

Comparable results were observed in a study from the western region of Saudi Arabia, which reported consistent findings regarding 30-day all-cause mortality, with about 47.8%. These results highlight the high fatality rate associated with *C. auris* infections among the Saudi population [39]. In Mexico, an even higher mortality rate of 83.3% was documented despite antifungal treatment, reflecting the growing concern of antifungal resistance among *C. auris* isolates [38]. Collectively, these findings underscore the urgent need for early detection and the timely initiation of effective antifungal therapy, as delays in diagnosis and treatment significantly increase the mortality risk [13].

Interestingly, in our cohort, 30-day mortality was lower among patients in the infection group. This finding likely reflects survival bias, as many patients developed infection later during their hospital stay. The median time from colonization to infection was approximately three weeks, and the median time to death among the infection group was 43 days, compared to 15 days in the colonization group. This suggests that patients who progressed to infection often survived beyond the initial 30-day period but ultimately experienced poor outcomes due to the cumulative burden of infection, comorbidities, and prolonged hospitalization. Therefore, 30-day mortality may not fully capture the long-term impact of *C. auris* infection, underscoring the importance of evaluating extended outcome measures in future studies.

This study has several limitations. The retrospective design may have introduced selection and information bias, as data were limited to what was available in medical records. Moreover, the retrospective design may have limited the accuracy of outcome assessment, particularly regarding events occurring after hospital discharge. Although we evaluated in-hospital mortality, post-discharge outcomes, including late infections or deaths, may have been under-detected. In addition, the relatively small number of patients who progressed to infection may limit the power to detect specific associations and preclude more detailed subgroup analyses. Antifungal susceptibility testing was not uniformly performed, limiting the ability to examine the impact of resistance patterns on outcomes. Furthermore, because of the limited sample size in the infection group (44 cases), we were unable to include all potentially relevant risk factors in the regression model, and unmeasured confounding cannot be excluded. We identified abdominal surgery as the only independent predictor of progression from colonization to infection. However, we could not specify the types of abdominal procedures performed or determine whether infection occurred at abdominal or extra-abdominal sites because these data were unavailable. Consequently, we could not analyze outcomes based on timing relative to surgery. Such specificity would be crucial for establishing causation rather than confounding by overall illness severity in the immediate postoperative period. Additionally, information on neutropenia and its duration was also not captured, and future studies should assess neutropenia as a potential risk factor in patients with *C. auris* colonization. Finally, as this was a single-center study, the findings may not be generalizable to other settings with different patient populations or infection control practices.

## 4. Materials and Methods

### 4.1. Study Design and Setting

This was a single-center retrospective case–control study conducted at our tertiary hospital. The study included adult patients (18 years or older) who were hospitalized and had confirmed *C. auris* colonization identified by swab or non-sterile site culture between January 2017 and June 2023. During the study period, standardized institutional protocols for *C. auris* screening, isolation, and infection control were applied, including reinforced hand hygiene, appropriate use of personal protective equipment, and enhanced environmental cleaning [40]. Patients were excluded if they had a positive *C. auris* culture before the swab collection or if the chart was missing key relevant data, such as clinical, microbiological, and laboratory data. Eligible patients were categorized into two groups: the cases (infection group), which included patients who progressed to invasive *C. auris* infection, and the controls (colonization group), which included patients who remained colonized without subsequent evidence of invasive infection.

We estimated the study sample size based on a study by Alaniazi et al., which reported a *C. auris* frequency of 56.9% in Saudi Arabia [29]. Thus, with a two-tailed test with a probability of error (alpha) of 5% and a power of 95%, the minimum sample size was 31 patients, with a critical t value of 2.04 and degrees of freedom of 29. The sample size calculation was performed on G*Power software version 3.1 (©2025 Heinrich-Heine-Universität Düsseldorf).

### 4.2. Ethical Approval

Ethical approval for this study was obtained from the Institutional Review Board of King Abdullah International Medical Research Center (KAIMRC) (Reference No. NRC22R/260/06). The research was conducted in accordance with the Declaration of Helsinki and its subsequent amendments. The Institutional Review Board granted a waiver of informed consent.

### 4.3. Definitions

*C. auris* colonization: Defined as the detection of *C. auris* in a surveillance swab (e.g., axilla, groin, rectum, or nasal cavity) or non-sterile site culture (e.g., urine or respiratory tract) without clinical signs or symptoms of infection.

Urinary colonization (asymptomatic candiduria): Defined as the isolation of *C. auris* from urine culture in the absence of urinary tract infection symptoms and without initiation of antifungal therapy.

Symptomatic UTI: Defined as the isolation of *C. auris* from urine culture in the presence of documented urinary tract infection symptoms or initiation of antifungal therapy, likened to a documented UTI diagnosis by the treating physician.

Invasive infection: Defined as isolation of *C. auris* from a normally sterile site (e.g., blood, cerebrospinal fluid, peritoneal fluid) in the presence of clinical signs or symptoms of infection.

Progression to invasive infection: Defined as the development of invasive *C. auris* infection within 90 days of the first documented colonization event (i.e., the date of the initial positive surveillance or non-sterile site culture). Infections occurring beyond this 90-day window are not considered progression from colonization.

Immunosuppressed status: Defined as the presence of any of the following: documented neutropenia, chronic treatment with corticosteroids, active chemotherapeutic management of malignancy, history of solid organ/bone marrow transplant, leukemia, AIDS, or lymphoma.

### 4.4. Outcome Measures

Primary outcome: Progression to invasive *C. auris* infection within 90 days of the first positive colonization swab.

Secondary outcomes: Risk factors associated with progression to invasive infection (e.g., comorbidities, immunosuppression, ICU admission, presence of invasive devices), receipt of antifungal therapy during hospitalization, length of hospital stay (from date of colonization), and 30-day all-cause mortality (calculated from the date of invasive infection for the infection group or from date of colonization for the colonization group).

### 4.5. Statistical Analysis

The normality of the data distribution was assessed visually using histograms. Categorical variables are presented as frequencies and percentages and were compared using either the Chi-square or Fisher’s exact test. Parametric continuous variables were presented as means and standard deviations (SD), while non-parametric continuous variables were presented as medians and interquartile ranges [IQR]. Continuous variables were compared using Student’s *t*-test and Mann–Whitney U test for parametric and non-parametric data, respectively. A two-sided *p*-value of <0.05 was considered statistically significant. The analyses were performed using Microsoft Excel version 16.93.1 (Microsoft Corporation, Redmond, WA, USA) and GraphPad Prism version 9.5.1 (GraphPad Software, Boston, MA, USA). Furthermore, we performed logistic regression analysis to identify risk factors with STATA 18 (Stata Corporation, College Station, TX, USA). Logistic regression analysis was reported as an odds ratio (OR) with a 95% confidence interval (CI).

## 5. Conclusions

Colonization with *C. auris* carries a measurable risk of progression to invasive disease. In our cohort, 13% of colonized patients developed infection, typically within three weeks of detection. Among several potential risk factors, recent abdominal surgery was the only independent predictor of progression, underscoring the importance of barrier disruption and postoperative vulnerability in facilitating fungal invasion. While the presence of invasive devices and critical illness was more common among infected patients, these factors were not independently associated with progression after adjustment for confounders, suggesting they reflect illness severity rather than direct causation. Patients who developed infection experienced significantly higher in-hospital mortality and prolonged hospital and ICU stays, highlighting the substantial clinical impact of *C. auris*. Early recognition of high-risk patients, particularly those undergoing abdominal surgery, paired with rapid notification, appropriate patient placement (single rooms or cohorting), and strict adherence to international (CDC, ECDC) and national (Saudi General Directorate for Infection Prevention and Control) guidance, is essential to limit transmission. Practical steps include enhanced screening of high-risk units, strict contact precautions, and environmental decontamination with agents effective against *C. auris*. Timely antifungal management and reporting to public health authorities further support early containment. Future research should explore optimized screening strategies and postoperative risk-reduction interventions to minimize progression from colonization to invasive disease.

## Figures and Tables

**Table 1:** Baseline characteristics of the study participants.

Variable	Colonization Group (n = 290)	Infection Group (n = 44)	Total (N = 334)	*p*-Value
Age, years, mean ± SD	66.13 ± 16.96	68.20 ± 15.61	66.4 ± 16.8	0.4467
Men, n (%)	166 (57.2)	24 (54.5)	190 (56)	0.7365
BMI, mean ± SD	30.3 ± 9.3	28.7 ± 8.4	30.1 ± 9.2	0.30
WBC count, mean ± SD	11.71 ± 6.75	11.46 ± 5.85	11.7 ± 6.6	0.8177
CRP, median (IQR)	65 (25–146)	65 (36–117)	65 (25–146)	0.9188
Procalcitonin, median (IQR)	0.63 (0.17–2.33)	0.475 (0.275–1.155)	0.61 (0.19–2.06)	0.876
Charlson Comorbidity Index, mean ± SD	4.79 ± 2.7	5.9 ± 3.1	4.9 ± 2.8	**0.0112**
Diabetes mellitus	168 (57.9)	30 (68.2)	198 (59.3)	0.1972
With complications, n (%)	25 (8.6)	2 (4.55)	27 (8.08)	0.3555
Without complications, n (%)	143 (49.3)	28 (63.6)	171 (51.2)	0.0765
Moderate to severe CKD, n (%)	66 (22.76)	11 (25)	77 (23.1)	0.7422
Malignancy	32 (11.03)	6 (13.64)	38 (11.38)	0.6125
Solid tumor, localized, n (%)	20 (6.9)	4 (9.09)	24 (7.19)	0.5994
Solid tumor, metastasized, n (%)	8 (2.76)	2 (4.55)	10 (2.99)	0.5170
Hematologic, n (%)	4 (1.38)	0 (0)	4 (1.2)	0.4332
Immunosuppression, n (%)	39 (13.45)	7 (15.91)	46 (13.77)	0.6589
Multi-site colonization, n (%)	137 (47.24)	24 (54.5)	161 (48.2)	0.3663
Source of colonization, n (%)				
Axilla	100 (34.48)	12 (27.27)	112 (33.53)	0.3452
Groin	114 (39.31)	14 (31.82)	128 (38.32)	0.3408
Rectal	43 (14.83)	11 (25.00)	54 (16.17)	0.0877
Nasal	25 (8.62)	6 (13.64)	31 (9.28)	0.2853
Urine	8 (2.76)	1 (2.27)	9 (2.69)	0.8529
ICU admission before colonization, n (%)	264 (91.0)	40 (90.9)	304 (91.0)	0.9784
Central line, n (%)	208 (71.7)	40 (90.9)	248 (74.25)	**0.0067**
Foley catheter, n (%)	204 (70.34)	37 (84.1)	241 (72.2)	0.0580
Mechanical ventilation, n (%)	175 (60.34)	34 (77.3)	209 (62.6)	**0.0306**
Antibiotic exposure (past 90 days), n (%)	260 (89.66)	42 (95.45)	302 (90.4)	0.2233
Duration of prior antibiotic use, days, median (IQR)	7 (3–10)	7 (4–11)	7 (3–10)	0.4121
Antibiotic used for ≥48 h, n (%)				
Ceftriaxone	15 (5.17)	0 (0)	15 (4.49)	0.1227
Linezolid	7 (2.41)	0 (0)	7 (2.1)	0.2976
Meropenem (Single agent)	82 (28.28)	7 (15.91)	89 (26.65)	0.0838
Meropenem (Combination)	7 (2.41)	1 (2.27)	8 (2.4)	0.9545
Piperacillin/Tazobactam	92 (31.7)	16 (36.36)	108 (32.34)	0.5398
Vancomycin	25 (8.62)	6 (13.6)	31 (9.28)	0.2853
Other *	32 (11.03)	14 (31.8)	46 (13.77)	**0.0002**
TPN within 30 days prior to colonization, n (%)	5 (1.72)	3 (6.82)	8 (2.4)	**0.0395**
Previous abdominal surgery within 30 days of colonization, n (%)	14 (4.83)	6 (13.64)	20 (5.99)	**0.0217**
Previous hospitalization within three months of colonization, n (%)	115 (39.66)	20 (45.45)	135 (40.4)	0.4651
Previous hospitalization within six months of colonization, n (%)	122 (42.1)	17 (38.64)	139 (41.6)	0.6669
HIV, n (%)	1 (0.34)	0 (0)	1 (0.3)	0.6965

*p*-values in bold indicate statistically significant differences (*p* < 0.05). SD: standard deviation, BMI: body mass index in kg/m^2^, CKD: chronic kidney disease. ICU: intensive care unit, IQR: interquartile range, TPN: total parenteral nutrition, HIV: human immunodeficiency virus. * Other antibiotics include amikacin, amoxicillin, amoxicillin/clavulanic acid, ampicillin/sulbactam, azithromycin, aztreonam, cefazolin, cefepime, ceftazidime, ceftazidime/avibactam, cefuroxime, ciprofloxacin, clindamycin, daptomycin, doxycycline, gentamicin, imipenem/cilastatin, metronidazole, tigecycline, and trimethoprim/sulfamethoxazole.

**Table 2:** Microbiological data of the infection group (n = 44).

Variable	Cohort
Days from colonization to a positive culture, median (IQR)	21 (12–35)
Source of positive *C. auris* culture, n (%)	
Urine	25 (56.82)
Blood	19 (43.18)
Echinocandins Resistance, n (%) *	
Anidulafungin	1/20 (5)
Caspofungin	3/20 (15)

* Antifungal susceptibility testing was performed on 20 isolates (45.45%). Note: *C. auris* typically shows resistance to azoles and polyenes [27,28]. We are reporting results for echinocandins, which are the recommended first-line agents. IQR: interquartile range, UTI: urinary tract infection.

**Table 3:** Clinical Outcomes Post *Candidozyma auris* Colonization.

Outcomes	Colonization Group (n = 290)	Infection Group (n = 44)	Odds Ratio (95% CI)	*p*-Value
In-hospital mortality, n (%)	96 (33.1)	29 (65.9)	3.91 (2.0–7.6)	**0.0001**
Mortality within 30 days of colonization, n (%)	69 (23.79)	11 (25)	1.07 (0.51–2.22)	0.8613
Time to death, days, median (IQR)	15 (5–40)	43 (25–88)	N/A	**0.000233**
Length of ICU stay, days, median (IQR)	17 (7–36)	30 (10–66)	N/A	**0.03196**
Length of hospital stay, days, median (IQR)	40 (20–83)	73 (43–147)	N/A	**0.000446**

*p*-values in bold indicate statistically significant differences (*p* < 0.05). IQR: interquartile range, ICU: intensive care unit, CI: confidence interval.

**Table 4:** Risk factors for progression from colonization to fungal infection.

Variable	Odds Ratio	95% Confidence Interval	*p*-Value
Central venous catheter	1.77	0.54–5.71	0.34
Mechanical ventilation	1.42	0.60–3.3	0.42
TPN within 30 days	1.45	0.22–9.14	0.7
Abdominal surgery within 30 days	4.08	1.1–15.12	**0.03**
In-hospital mortality	7.8	3.28–18.5	**<0.001**
30-day mortality	0.27	0.11–0.66	**0.005**

*p*-values in bold indicate statistically significant differences (*p* < 0.05). TPN: total parenteral nutrition.

## Data Availability

The datasets generated during and/or analyzed during the current study are available from the corresponding author on reasonable request.
